# Case Report: Colorectal cancer metastasis to a cervical lymph node – an unusual source of a neck lump

**DOI:** 10.12688/f1000research.22560.1

**Published:** 2020-04-17

**Authors:** Oliver J Wright, Anthony Bashyam, Lisa Pitkin, Silvana Di Palma

**Affiliations:** 1 Otolaryngology Department, Frimley Park Hospital, Portsmouth Rd, Frimley, GU16 7UJ, UK; 2 Royal Surrey County Hospital NHS Foundation Trust, Guildford, GU2 7XX, UK

**Keywords:** Colorectal cancer, neck lump, Cervical lymph node, metastatic caecal adenocarcinoma, otolaryngology

## Abstract

Colorectal cancer (CRC) is the third most common cancer worldwide, and approximately 25% of patients already have metastases at the time of diagnosis. The most common metastatic sites for CRCs are the liver, lung, bone and brain and peritoneum. Cervical lymph node metastases in CRC are rare, particularly in the absence solid organ involvement. Here we present a case of a 73-year-old female patient who, following resection of a poorly differentiated caecal adenocarcinoma, re-presented four years later with a left level IV lymph node which was ultimately found to contain metastatic adenocarcinoma.

## Introduction

Cervical lymphadenopathy refers to the pathological enlargement of cervical lymph nodes. Generally, it is reactive, benign and self-limiting, but in some patients is a presenting sign of an occult malignancy.

Malignant lymphadenopathy may be primary (Hodgkin lymphoma, Non-Hodgkin lymphoma) or metastatic (squamous cell carcinomas of the skin and upper aerodigestive tract or salivary/thyroid gland carcinomas). Metastatic spread is typically along lymphatic channels from the head and neck, but in 1% of cases originates more distally (most commonly from the breast, lung, kidney, tests and cervix) ^1^.

Colorectal cancer accounts for 10.2% of cancer incidence worldwide, with nearly two million cases diagnosed in 2018 and makes up 9.2% (881,000) of all cancer-related deaths ^2^.

As with many malignancies, the development of metastatic disease is associated with a poorer prognosis. Approximately 25% of patients with colorectal cancer will have metastases at the time of diagnosis, and approximately half will go on to develop them ^3^. Colorectal malignancy can spread distally via lymphatic or haematogenous routes and typically metastasises to the liver, lung, bone, brain and peritoneum ^4^.

Here we present the case of a patient presenting with a neck lump that was ultimately found to be a metastatic caecal adenocarcinoma. The case describes an unusual pattern of disease spread, both anatomically and by histological subtype. The presence of cervical nodal disease in the absence of solid organ metastasis is particularly unusual.

## Case presentation

A 73-year-old female patient presented with small bowel obstruction and underwent an emergency right hemicolectomy for a caecal mass.

The patient had multiple comorbidities: chronic obstructive pulmonary disease, hypertension, deep vein thrombosis, non-ST-elevation myocardial infection, coronary artery bypass graft, osteoarthritis and a hiatus hernia. The patient was a current smoker with a 13-pack year history and minimal alcohol consumption.

Post-operative histology described a poorly differentiated caecal adenocarcinoma with clear resection margins and 4/20 regional nodes involved (TNM staging, T4N2M0). The colorectal multidisciplinary team (MDT) meeting recommended adjuvant chemotherapy to reduce the risk of cancer recurrence and treat any micro-metastatic disease. The recommendation was to complete eight cycles of capecitabine (130mg/m ^2^) and oxaliplatin (1000 mg/m ^2^ twice daily) chemotherapy over six months. Unfortunately, the patient only completed one cycle, declining further treatment due to the severity of side effects.

At one-year follow up, colonoscopy and a computerised tomography (CT) abdomen demonstrated no evidence of recurrent disease. Three years later, on surveillance imaging, para-aortic lymph node metastases were detected on positron emission tomography (PET) scanning. The patient underwent external beam para-aortic radiotherapy 50.4 Gy in 28 fractions, which was well tolerated, and was discharged to regular surveillance.

Three years later, a rapidly enlarging painless left-sided neck lump was detected. There was no dysphagia, odynophagia, dysphonia, loss of appetite or weight loss. On examination, there was a palpable small level IV neck lump which was smooth, non-fluctuant and non-tethered to underlying structures.

### Investigations

Routine blood tests, including full blood count, liver function and renal function, were unremarkable. Carcinoembryonic antigen, a tumour marker for colorectal carcinoma, was elevated at 24.0 mg/L (normal range <2.5 mg/L).

Ultrasound of the lump showed a 6-mm level IV cervical lymph node with intranodal calcifications, loss of hilar architecture and increased vascularity suspicious for malignancy.

Cytology from fine-needle aspiration of the node under ultrasound guidance showed clusters of atypical cells with pleomorphic nuclei and a moderate amount of cytoplasm. Immunohistochemical analysis of the cell block preparation showed cells which were positive for cytokeratin AE1/AE3 and CDX2. AE1/AE3 is an antibody-based assay which reacts with epithelial based tissues, and CDX2 is an intestine-specific transcription factor that is highly sensitive and specific for adenocarcinomas of intestinal origin ^5^.

**Figure 1.**
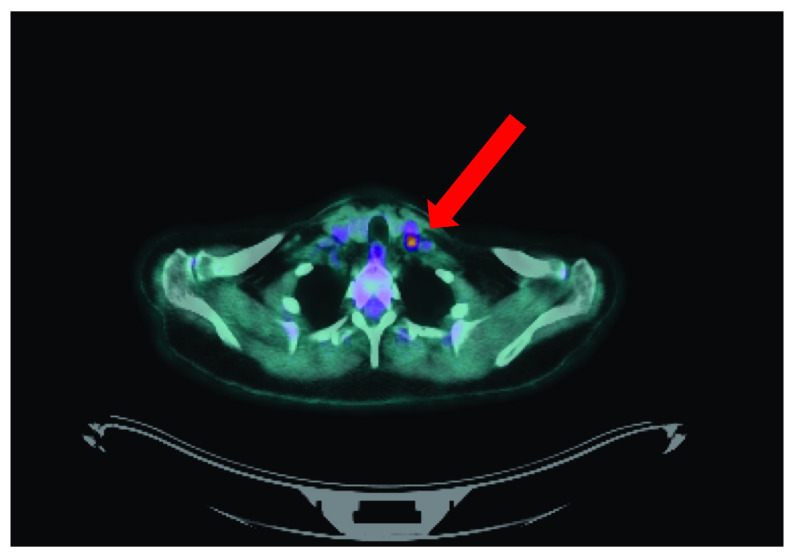
Positron emission tomography/computerised tomography demonstrating elevated tracer uptake in an 8.4-mm left level IV lymph node (red arrow).

Whole-body PET-CT showed elevated tracer uptake in an 8.4mm left level IV LN, and in a second 7-mm left supraclavicular lymph node with no further evidence of thoracic or abdominal recurrent disease (See Figure 1).

### Differential diagnosis

In the post-cancer-treatment surveillance context of this patient, any new, persistent lymphadenopathy should stimulate a high degree of diagnostic suspicion and investigations should be suitably intensive.

In the general population, the considered differential is broader. A ‘Surgical Sieve’ approach is useful in diagnosing a neck lump. The authors find the mnemonic VITAMIN-C particularly helpful (see Table 1).

**Table 1.** ‘Surgical sieve’ mnemonic for a neck lump.

Mnemonic letter	Symptom
V: vascular	Carotid body tumour, carotid aneurysm, glomus jugulare
I: infective	Reactive lymphadenopathy, tuberculosis, sialadenitis, dental abscess
T: traumatic	Haematoma
A: autoimmune	Thyroid goitre (Grave’s disease, Hashimoto’s thyroiditis)
M: metabolic	Thyroid goitre (Iodine deficiency), Hyperparathyroidism and salivary calculus
I: inflammatory	Sarcoidosis
N: neoplastic	Lymphoma, Squamous Cell Carcinoma, salivary neoplasm, thyroid neoplasm, lipoma, metastatic disease
C: congenital	Cystic hygroma, thyroglossal duct cysts, branchial cyst, dermoid cyst, teratoma, thymic cysts

Following a thorough history and examination, the principal method of investigating a neck lump is through ultrasound imaging and if suspicious, a fine-needle aspiration or core biopsy.

Reactive lymph nodes have a different ultrasound appearance to malignant ones which are typically larger and more spherical in shape. An irregular border indicating extracapsular spread, hypo-echogenicity, macrocalcifications, intranodal necrosis and peripheral vascularity are all pathological features concerning for malignant disease ^6^. Further imaging with CT, PET-CT, or MRI is useful in defining the lesion, staging disease and in radiotherapy planning.

### Treatment

On day 35 following re-presentation, the patient was discussed at the Head and Neck Cancer MDT in conjunction with the Colorectal Cancer MDT, the recommendation was made for a selective neck dissection of levels 2, 3, 4, and 5. The surgery went without complication, and the patient was discharged the following day.

**Figure 2.**
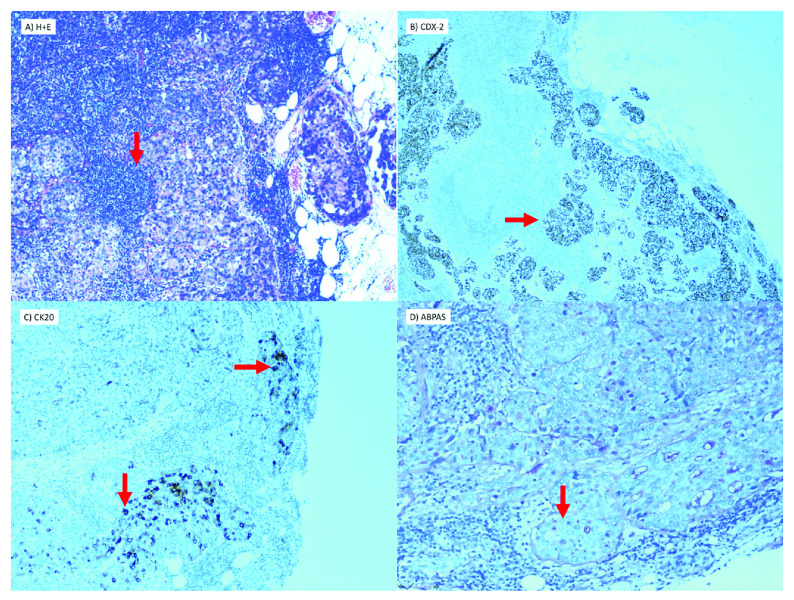
Histology from left neck dissection showing metastasis of poorly differentiated carcinoma in lymph node (40X magnification). ( **a**) Atypical cells with pleomorphic nuclei demonstrated Hematoxylin and Eosin stain. ( **b**) Immunohistochemistry for CK20 with characteristic cytoplasmic and nuclear staining. ( **c**) Immunohistochemistry for CDX-2. ( **d**) Intracellular mucus detected on special stain (ABPAS) confirming glandular (adenocarcinoma). ABPAS is an Alcian Blue stain which demonstrates intra and extracellular mucous.

Histology from the dissection showed 6/20 lymph nodes with metastatic deposits of poorly differentiated adenocarcinoma. There was with evidence of intracellular mucous and positive staining for CK20 and CDX-2. Immunohistochemical analysis confirmed an intestinal origin ( Figure 2).

### Outcome and follow-up

The patient recovered well from the neck dissection, and at two-month review, the patient was disease-free and asymptomatic.

At two-year review, the patient had developed some intermittent abdominal discomfort. CT imaging revealed small volume retroperitoneal and pelvic lymphadenopathy. Since she had previously received radiotherapy to the area, the oncology team felt that the only treatment they would recommend would be chemotherapy; however, the patient declined because of side-effects and her comorbidities. She remains under three-monthly surveillance in the outpatient department.

## Discussion

Pathological cervical lymphadenopathy is most commonly due to metastasis from malignancies that originate in the head and neck. Common primary sites include skin cancers, salivary gland tumours, thyroid carcinomas and squamous cell carcinomas of the upper aerodigestive tract ^1^.

Generally, malignancies metastasise along predictable routes of lymphatic drainage to nodal areas, which has led to the development of neck levels (I-VII) that guide surgical management. Investigations can be targeted to the likely primary site based on the location of the lymphadenopathy; for example, level I nodes drain the oral cavity, submandibular gland, lip and anterior nasal cavity.

The presentation of metastasis from more distant sites, particularly infraclavicular, is more challenging to account for as there is often no apparent lymphatic route. A potential mechanism of spread is via obstruction of the thoracic duct.

The thoracic duct is the largest lymphatic vessel in the body. It extends from the cisterna chyli at the level of the second lumbar vertebrae and ascends through the abdomen and thorax to terminate into the left subclavian vein. It receives afferents from the thorax, abdomen and pelvis, and therefore provides a potential route for the spread of disease to left supraclavicular lymph node. Virchow’s node is the eponymous name given to this node, and Trosier’s sign refers to it pathological enlargement, typically due to gastric or pulmonary malignancy ^7^. Lymphatic drainage of the head and the neck is via the superficial and deep lymphatic vessels which converge to form the right and left jugular lymphatic trunks. Vessels from the right terminate in the right lymphatic duct and those on the left, in the thoracic duct. Lymphatic obstruction at supraclavicular or lower cervical nodes can, therefore, result in the retrograde spread of abdominal or thoracic disease to higher cervical or even contralateral lymph nodes ^1^.

Different histological subtypes of colorectal cancer also appear to have predilections for distinct metastatic sites. In an autopsy study of 1675 patients with metastatic disease, Hugen *et al*. ^8^ found that adenocarcinomas preferentially metastasised to the liver, while mucinous and signet ring cell carcinomas were more frequently associated with peritoneal metastasis. Signet ring cell carcinomas metastasised to distant lymph nodes more frequently than adenocarcinomas and mucinous carcinomas. Interestingly, there appeared to be no significant difference in the rate of pulmonary metastasis between histological subtypes ^8^.

The study also demonstrated a difference in metastases distribution based on the original tumour site. While both primarily metastasised to the liver, colonic malignancies were more likely to present with intra-abdominal metastasis (peritoneal, omental or ovarian) and rectal cancers were more frequent in extra-abdominal sites (lung, brain). The venous drainage of the rectum can explain its more wandering metastatic spread. While the superior and inferior mesenteric veins drain the colon and upper two-thirds of the rectum into the portal venous system; the lower third of the rectum is drained by the middle and inferior rectal veins which bypass the liver and directly enter the inferior vena cava. Our case is therefore made more unusual by the fact it was neither rectal or a signet ring cell carcinoma; so distant cervical metastasis is unexpected. In patients with only one distant metastasis, the site of metastases also appears to be an important prognostic factor. For example, thoracic metastases are associated with longer median survival than peritoneal, bone or nervous system metastases ^9^.

An understanding of the different patterns of metastatic spread between subtypes is of clinical importance as it may affect both immediate management and follow up techniques. In line with guidance from the National Institute for Health and Care Excellence, patients routinely undergo at least two CT scans of the chest, abdomen pelvis in the first three years after treatment ^10^. Given the findings of Hugen *et al*., a case could be made for imaging additional sites or using other imaging modalities such as PET-CT in higher-risk subtypes such as signet ring cell or mucinous carcinoma.

Several case reports have been published on the metastatic spread of colorectal carcinoma to the thyroid gland ^11– 13^, but isolated metastatic spread to cervical lymph nodes is rare, and treatment decisions are made on a case by case basis. Aksel *et al*. ^4^ reported on a case of cervical lymphadenopathy as the presenting feature of undiagnosed sigmoid colon carcinoma. The patient was treated with simultaneous resection of the colonic primary and left neck node dissection. Postoperatively the patient received adjuvant folinic acid/fluoronacil/oxaliplatin chemotherapy and was disease-free at nine-month follow up ^4^. Ochi *et al*. ^14^ reported on a case of metastatic recurrent sigmoid colon cancer to Virchow’s lymph node. This was managed non surgically with systemic chemotherapy and radiotherapy, which achieved a complete response and no recurrence at the ten-month follow-up ^14^.

In conclusion, although most metastatic lymphadenopathy originates from head and neck primaries, clinicians should always consider the possibility of more remote disease and not underestimate the importance of a detailed past medical history and systems review. Management of metastatic disease to the neck should be discussed by the relevant cancer MDTs and may involve surgical resection and chemotherapy with or without radiotherapy.

## Learning points/take home messages

1. Most metastatic neck nodes arise from primary tumours of the head and neck
2. Occasionally the metastases are from intra-abdominal malignancies, most commonly breast, lung, kidney and rarely bowel.
3. A thorough history and examination is essential in assessing a patient with a neck lump
4. Management of metastatic disease to the neck is on a case by case basis in conjunction with the Multi-Disciplinary Team discussion

## Data availability

All data underlying the results are available as part of the article and no additional source data are required.

## Consent

Written informed consent for publication of clinical details and clinical images was obtained from the patient.

## References

[1] López F Rodrigo JP Silver CE: Cervical lymph node metastases from remote primary tumor sites. *Head Neck.* 2016;38(Suppl 1):E2374–E2385. 10.1002/hed.24344 26713674 PMC4991634

[2] WHO Factsheet.2018; Accessed: 29/9/2019. Reference Source

[3] Vatandoust S Price TJ Karapetis CS: Colorectal cancer: Metastases to a single organ. *World J Gastroenterol.* 2015;21(41):11767–11776. 10.3748/wjg.v21.i41.11767 26557001 PMC4631975

[4] Aksel B Dogan L Karaman N: Cervical Lymphadenopathy as the First Presentation of Sigmoid Colon Cancer Case Report. *Middle East Journal of Cancer.* 2013;4(4):185–188. Reference Source

[5] Werling RW Yaziji H Bacchi CE: CDX2, a highly sensitive and specific marker of adenocarcinomas of intestinal origin: an immunohistochemical survey of 476 primary and metastatic carcinomas. *Am J Surg Pathol.* 2003;27(3):303–310. 10.1097/00000478-200303000-00003 12604886

[6] Ahuja AT Ying M Ho SY: Ultrasound of malignant cervical lymph nodes. *Cancer Imaging.* 2008;8(1):48–56. 10.1102/1470-7330.2008.0006 18390388 PMC2324368

[7] Zdilla MJ Aldawood AM Plata A: Troisier sign and Virchow node: the anatomy and pathology of pulmonary adenocarcinoma metastasis to a supraclavicular lymph node. *Autopsy Case Rep.* 2019;9(1):e2018053. 10.4322/acr.2018.053 30863728 PMC6394356

[8] Hugen N van de Velde CJ de Wilt JH: Metastatic pattern in colorectal cancer is strongly influenced by histological subtype. *Ann Oncol.* 2014;25(3):651–657. 10.1093/annonc/mdt591 24504447 PMC4433523

[9] Riihimaki M Hemminki A Sundquist J: Patterns of metastasis in colon and rectal cancer. *Sci Rep.* 2016;6: 29765. 10.1038/srep29765 27416752 PMC4945942

[10] NICE: Colorectal cancer: diagnosis and management Clinical guideline [CG131]. Published date: November 2011; Last updated: December 2014; Accessed: 29/9/2019. Reference Source

[11] Coelho MI Albano MN Costa Almeida CE: Colon cancer metastasis to the thyroid gland: A case report. *Int J Surg Case Rep.* 2017;37:221–224. 10.1016/j.ijscr.2017.06.035 28709052 PMC5508494

[12] Akimaru K Onda M Tajiri T: Colonic adenocarcinoma metastatic to the thyroid: report of a case. *Surg Today.* 2002;32(2):151–4. 10.1007/s005950200009 11998944

[13] Minami S Inoue K Irie J: Metastasis of colon cancer to the thyroid and cervical lymph nodes: a case report. *Surg Case Rep.* 2016;2(1): 108. 10.1186/s40792-016-0237-3 27714647 PMC5053968

[14] Ohchi T Akagi Y Kinugasa T: Virchow lymph node metastatic recurrence of sigmoid colon cancer with severe lymph node metastases successfully treated using systemic chemotherapy combined with radiotherapy. *Anticancer Res.* 2013;33(7):2935–40. 23780983

