# Supplementary material for: Case Report: Colorectal cancer metastasis to a cervical lymph node – an unusual source of a neck lump
Source: F1000Res. 2020 Apr 17;9:266. [Version 1] doi: 10.12688/f1000research.22560.1 (PMC7845154; doi:10.12688/f1000research.22560.1)
Supplement: Peer review history

# Peer review: Reviewer response for version 1

**DOI:** 10.5256/f1000research.24902.r74782

**Published:** 2021-01-27

**Authors:** Thomas Geyton

**Affiliations:** 1 Basingstoke and North Hampshire Hospital, Basingstoke, UK

This ia a well written interesting article which describes the presentation of a 73 year old patient with a neck lump. Key features of the medical history, imaging and histology are explored and the case described the rare event of cervical metastasis to a cervical lymph node. The authors explore the anatomical background to this presentation and contextualise in relation to the current literature. 

 I found this article interesting, scientifically sound and coherent. I therefore have no reservations in regard to its indexing in its current form. 

Are enough details provided of any physical examination and diagnostic tests, treatment given and outcomes?

Yes

Is the case presented with sufficient detail to be useful for other practitioners?

Yes

Is sufficient discussion included of the importance of the findings and their relevance to future understanding of disease processes, diagnosis or treatment?

Yes

Is the background of the case’s history and progression described in sufficient detail?

Yes

Reviewer Expertise:

ENT

I confirm that I have read this submission and believe that I have an appropriate level of expertise to confirm that it is of an acceptable scientific standard.

# Peer review: Reviewer response for version 1

**DOI:** 10.5256/f1000research.24902.r62874

**Published:** 2020-05-26

**Authors:** Carlos Manuel Costa Almeida

**Affiliations:** 1 Faculty of Medicine of the University of Coimbra, Coimbra, Portugal; 2 Centro Cirúrgico de Coimbra, Coimbra, Portugal

Globally, I think this paper can be indexed, but I have to make a few remarks on some aspects that I would like the authors to address in the paper before its indexed.

1. When the patient had the primary colon tumour, how was the CEA? It is not registered then, we only know that it was elevated when the cervical mass was noted, three years later.
2. What about colonoscopy? Was it done any time during the three years of follow-up? Or not? And in a cervical mass, apparently a metastatic lymph node, in a patient who had a colon cancer three years before, shouldn’t it be done as part of the patient study?
3. How was the follow-up of the primary tumour done? And how should it be done? This would be a valuable piece of information for this case and its report.
4. A cervical lymphatic metastasis in the left supraclavicular notch can originate virtually anywhere below the diaphragm, in the abdominal cavity and the testis, following the thoracic duct, as it is said. Besides the head and neck, and the left thorax, of course. The Virchow’s node and the Troisier’s sign are very appropriately referred to in the text. Such a metastasis coming from the colon or the rectum is not common, but it is not “unexpected”…

I am sure we agree that the colo-rectal cancer prognosis improved a great deal combining an appropriate surgical treatment with an efficacious chemotherapy. It was a pity the patient did not accept chemotherapy twice.

 Anyway, I think this is the report of an interesting case, that should be indexed, giving further information on supraclavicular metastization.

Are enough details provided of any physical examination and diagnostic tests, treatment given and outcomes?

Partly

Is the case presented with sufficient detail to be useful for other practitioners?

Yes

Is sufficient discussion included of the importance of the findings and their relevance to future understanding of disease processes, diagnosis or treatment?

Yes

Is the background of the case’s history and progression described in sufficient detail?

Partly

Reviewer Expertise:

General Surgery.

I confirm that I have read this submission and believe that I have an appropriate level of expertise to confirm that it is of an acceptable scientific standard, however I have significant reservations, as outlined above.

# Peer review: Reviewer response for version 1

**DOI:** 10.5256/f1000research.24902.r62875

**Published:** 2020-05-04

**Authors:** Alfio Ferlito

**Affiliations:** 1 International Head and Neck Scientific Group, Padua, Italy

The manuscript is accepted in its current form. This is an interesting and well written paper. The radiological and histological documentation is adequately represented in support of the case report. Metastatic cancer in the cervical region is always a challenge to the head and neck specialist, in particular if the primary tumour site is unknown. This paper can be useful especially to a young readership of head and neck surgeons/trainees.

Are enough details provided of any physical examination and diagnostic tests, treatment given and outcomes?

Yes

Is the case presented with sufficient detail to be useful for other practitioners?

Yes

Is sufficient discussion included of the importance of the findings and their relevance to future understanding of disease processes, diagnosis or treatment?

Yes

Is the background of the case’s history and progression described in sufficient detail?

Yes

Reviewer Expertise:

Head and neck surgery/oncology

I confirm that I have read this submission and believe that I have an appropriate level of expertise to confirm that it is of an acceptable scientific standard.
